# A Dose-Effect Study of Cisplatin Ototoxicity in Albino Guinea Pigs

**DOI:** 10.22038/ijorl.2019.41914.2368

**Published:** 2020-05

**Authors:** Negin Salehi, Mehdi Akbari, Akram Pourbakht, Hamid Haghani, Mahyar Janahmadi

**Affiliations:** 1 *Department of Audiology, School of Rehabilitation Sciences, Iran University of Medical Sciences, Tehran, Iran.*; 2 *School of Nursing and Midwifery, Iran University of Medical Sciences, Tehran, Iran.*; 3 *Neuroscience Research Center and Department of Physiology, School of Medicine, Shahid Beheshti University of Medical Sciences, Tehran, Iran.*

**Keywords:** Auditory Brainstem Response (ABR), Cisplatin, Guinea pigs, Hearing loss, Ototoxicity

## Abstract

**Introduction:**

Cisplatin is one of the most commonly used antineoplastic drugs; nonetheless, its ototoxic dose-limiting side effects have remained a significant challenge in clinical practice. The recognition of the exact template of hearing loss induced by multiple low doses of cisplatin could be of great help in managing the treatment process. The present study aimed to investigate the effects of multiple doses of this drug on the auditory system.

**Material and Methods:**

The present study was performed using an experimental guinea pig model in four groups as follows:1- 0.9% sodium chloride solution, 2- total dose of 7.5 mg/kg Cisplatin, 3- total dose of 10 mg/kg Cisplatin, and 4- total dose of 12.5 mg/kg cisplatin. The drugs were injected as 2.5 mg/kg/daily IP access in all groups. The auditory brainstem response (ABR) test was performed before the treatment and after every injection on a daily basis up to 72 h after the last injection.

**Results:**

There was dose-dependent significant hearing loss in all evaluated frequencies in three cisplatin groups. The general template of induced hearing loss during experimental days was almost the same in groups Cis7.5 and Cis10. In Cis 12.5 group, there was a jump in the threshold shift on the 5th day of the experiment and an upward trend in the function.

**Conclusion:**

As evidenced by the obtained results, the monitoring of hearing loss after every injection in patients who receive the drug and detecting the exact dose-dependent pattern of the induced hearing loss is of great help in controlling its undesirable destructive side effects on the auditory system.

## Introduction

American Chemist, Barnett Rosenberg, in 1965 proved that certain platinum-containing compounds inhibit cell division and make considerable changes to bacterial morphology (1). The antineoplastic property of this newly discovered drug which was called cisplatin resulted in experimental studies and it eventually received food and drug administration (FDA) approval in 1978 (2). Nowadays it is a widely used anti-cancer drug in multiple cancers, such as testicular cancer and ovarian cancer. The recovery rate of this drug in testicular cancers is higher than 90% (3). Deoxyribonucleic acid (DNA) damage in proliferating cells is considered to be the primary underlying cause of the efficacy of this drug in fighting against tumors. Although the rate of cell division in most body cells is low or even zero, some cells are at higher risk of cisplatin-induced toxicity (4,5). This leads to serious side effects, such as ototoxicity, nephrotoxicity, and neurotoxicity (6-8). Ototoxicity is one of the widely recognized side effects of cisplatin treatment. Destruction of outer hair cells (OHCs) in the organ of Corti by apoptosis is the common mechanism of cisplatin ototoxicity (9). This process is accomplished by the production of free radicals, depletion of glutathione, and finally lipid peroxidation. On the other hand, reactive oxygen species (ROS) play a key role in cisplatin ototoxicity (10). 

Ototoxicity is manifested as bilateral progressive and irreversible sensory neural hearing loss in most cases (11). More than 60% of patients who receive this drug experience irreversible hearing loss which exerts a destructive impact on their educational and psychosocial development (12,13). 

Furthermore, the economic burden of hearing loss on society is the leading cause of the increasing necessity for finding an applicable method for the protection of cochlea against cisplatin ototoxicity(14). 

One of the principal considerations in the development of conservative methods is preventing cisplatin ototoxicity without disturbing its anticancer function (15). The prevailing suggested and investigated methods, such as multiple antioxidants, inhibition of cell death route, and the inhibition of inflammation, have numerous limitations in clinical practice (16-23). They are regarded as invasive methods in clinical practice due to the necessity of local application to the cochlea or interact with the drug in some cases leading to decreased antitumor efficacy (24,25). 

Accordingly, the audiological monitoring of patients and exerting limitations on drug dosage is considered the most appropriate method for the protection of cochlea against cisplatin-induced ototoxicity. The physician must be aware of early signs of ototoxicity and its progression and could manage to change the treatment method if necessary. Therefore, the importance of dose-dependent changes in the hearing system by cisplatin treatment has been highlighted in the literature. With this background in mind, the present study aimed to daily check the impact of multiple dosages of cisplatin on the hearing system of guinea pigs by auditory brainstem response.

## Material and Methods

In the present study, albino guinea pigs were selected as the laboratory animals for a few reasons. They are easy to handle and anesthetic infusion and the intraperitoneal (IP) injection of the drug are effortless in these animals; therefore, they are more sensitive to the effects of cisplatin. Animals were obtained from Razi Vaccine and Serum Research Institute consisting of 24 albino male guinea pigs within the weight range of 250-300 grams. Animals were kept under standardized housing and feeding conditions in separate cages. All of them had free access to commercial food and water and were maintained in an environment with a controlled temperature of 20-25 degrees Celsius and 12 h light-dark cycle. The protocol was approved by the Institutional Review Board, and the animals were handled in accordance with the guidelines proposed by the Animal Use Committee of the institution. After a 24-hour rest and confirming the positive Preyer’s reflex in all animals, guinea pigs were assigned to four treatment groups (n=6 each) as follows:

1. The daily IP administration of 0.9% sodium chloride solution 2.5 ml/kg 

2. The daily IP administration of cisplatin (1mg/ml) 2.5 mg/kg for three consecutive days (total dose of 7.5 mg/kg) (Cis7.5)

3. The daily administration of cisplatin (1mg/ml) 2.5 mg/kg for four consecutive days (total dose of 10 mg/kg) (Cis10)

4. The daily IP administration of cisplatin (1mg/ml) 2.5 mg/kg for five consecutive days (total dose of 12.5 mg/kg) (Cis12.5)

For the controlled application of drugs, we used 1 cc disposable insulin syringe for each animal.

Auditory Brainstem Response (ABR) test was performed using an electrophysiologic system (Biologic/Navigator pro, USA). The test was carried out before the treatment (baseline measurement) and after every injection on a daily basis. The injections were daily performed at 7 am and the hearing assessments were carried out at 7 pm. The hearing evaluation was continued up to 72 h after the last injection. The guinea pigs were anesthetized by intraperitoneal injection of a mixture of ketamine 40 mg/kg and xylazine 4 mg/kg. While ABR tests were performed, the body temperature was maintained at 36°C with an electric blanket controlled by a rectal thermistor. In the pilot study, it was confirmed that cisplatin induces bilateral and symmetrical hearing loss. Consequently, it was required to avoid data duplication and consider inter-subject variability factors. In this regard, the test was performed using a far-field technique from the right ears of all animals, and the left ear was blocked by an earplug during the test. The reference electrode was subcutaneously inserted into the ipsilateral pinna, the ground electrode into the contralateral pinna, and the active electrode into the top of the head. Before the commencement of the test, the impedance of electrodes was confirmed to be below 5 k Ohms. Acoustic stimuli were delivered by a far-field speaker in a sound-proof box, and the sound levels within the animal hearing range were calibrated with a sound level meter. Acoustic stimuli consisted of tone-bursts of 4, 8, 12, and 16 kHz (the total duration was 10 ms, and the rise and fall times were 2 ms). The result of the time window analysis for each response was 10.66 ms. The evoked potentials were filtered with a band-pass filter between 100 and 3000 Hz. The stimulus presentation rate was 23.1 bursts of alternating polarity per second. The minimum number of sweeps was reported as 1024, and the gain was adjusted at 100k.In evaluating the hearing thresholds, the stimuli levels were lowered from 80 in 5-dB steps until the identification of a visible repeatable wave III. The recordings were repeated twice at the threshold level for confirming the reproducibility of the waves. Wave III was used for the detection of thresholds.

The obtained data were analyzed in SPSS software (version 17) using repeated measures. The quantitative data were presented as mean±SD. A p-value less than 0.05 was considered statistically significant.

## Results

**Fig 1:**
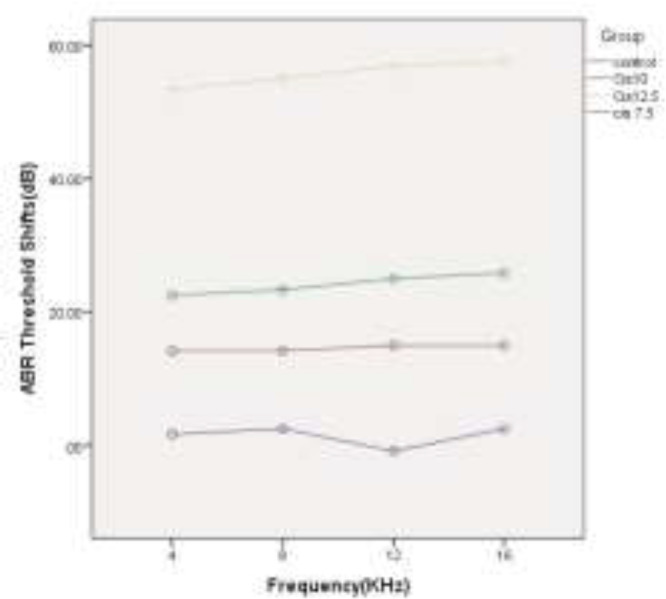
Mean auditory brainstem response threshold shifts (dB) in different frequencies in four experimental groups

The total change in ABR thresholds (last measurement-baseline measurement) in four different groups are displayed in Figure1. In all groups, cisplatin induced significant changes in ABR thresholds at all frequencies. The mean threshold shifts (Mean±SD) in group Cis7.5 were measured at 14.16±7.35 dB at 4 kHz (P=0.005), 14.21±7.20 dB at 8KHz(P=0.005), 15.00±7.07 dB at 12 kHz(P=0.003), and 15.24±7.02 dB at 16 kHz (p=0.003). In group Cis10, the mean threshold shifts were obtained at 22.50±6.89 dB (P=0.000) in 4 kHz, 23.33±6.83 dB (P=0.000) in 8 kHz, 25.00±10.00 dB (P=0.002) in 12 kHz, and 25.83±5.84 dB (P=0.000) in 16 kHz. The mean ABR threshold shifts in group Cis12.5 were calculated at 55.33±6.83 dB (P=0.000) in 4 kHz, 55.00±5.47 dB (P=0.000) in 8 kHz, 56.83±4.91 dB (P=0.000) in 12 kHz, and 57.50±4.18 dB (P=0.000) in 16 kHz. In the control group, no significant change was observed in mean ABR threshold shifts between the last measurement and the baseline values. The changes were 1.66±2.58 dB (P=0.175) in 4 kHz, 2.50±6.89 dB (P=0.415) in 8 kHz, -0.83±6.64 dB(P=0.771) in 12 kHz, and 2.50±5.24 dB(P=0.296) in 16 kHz.

**Fig 2:**
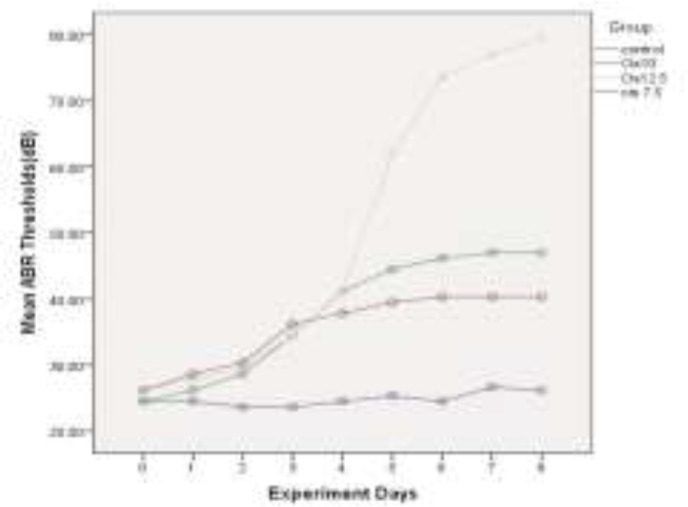
Mean auditory brainstem response thresholds (dB) in experimental days in 4kHz

**Fig 3:**
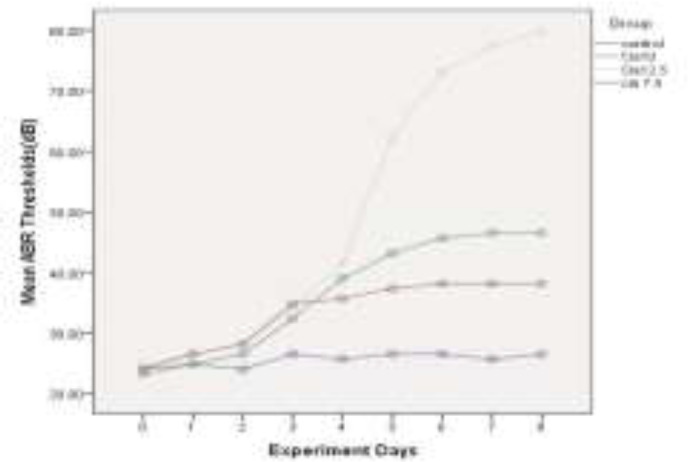
Mean auditory brainstem response thresholds(dB) in experimental days in 8kHz

**Fig 4:**
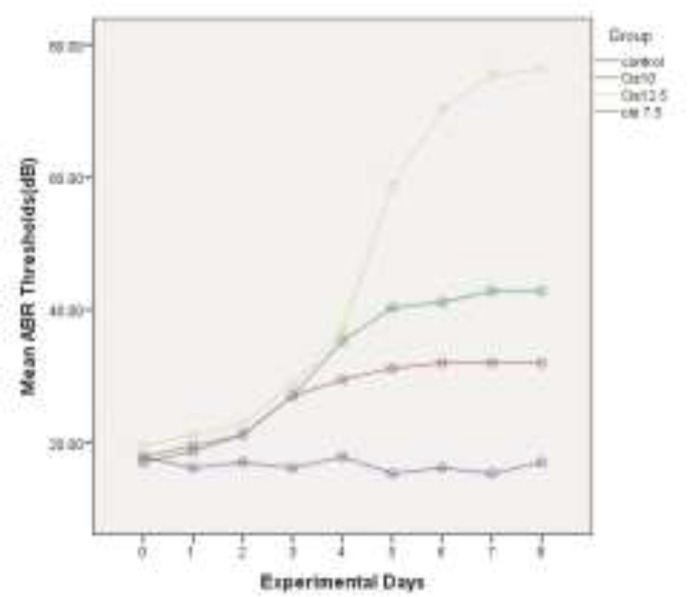
Mean auditory brainstem response thresholds (dB) in experimental days in 12 kHz

**Fig 5:**
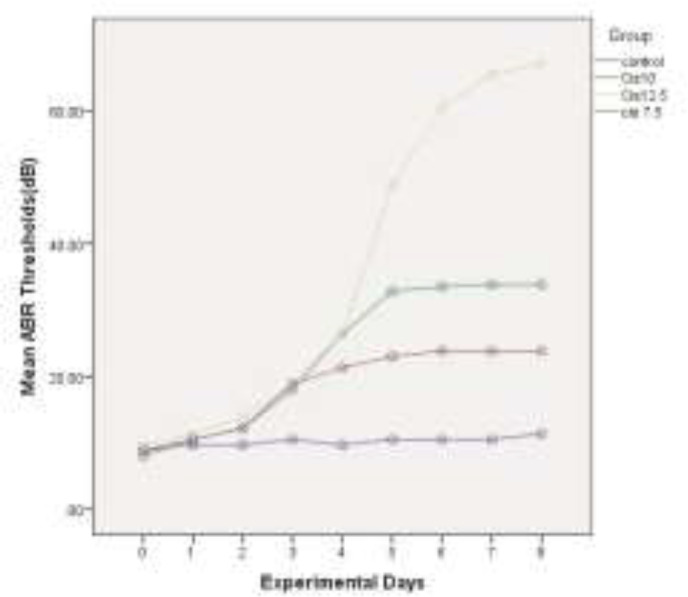
Mean auditory brainstem response thresholds (dB) in experimental days in 16 kHz

The mean of ABR thresholds(dB) within 8 experiment days for all four groups is depicted in figures 2, 3, 4 and 5, for 4 kHz, 8 kHz, 12 kHz, and 16 kHz tones, respectively. The changes in ABR thresholds during the experiment days are statistically significant in all groups which received cisplatin (Cis7.5, Cis10, and Cis12.5) (Table.1).

**Table1:** Mean auditory brainstem response thresholds (dB) for 4 kHz, 8 kHz, 12 kHz, and 16 kHz tones in all groups in 8 experiment days

frequency			**Mean auditory brainstem response thresholds(dB)±SD**									P-value
			**Experiment days**									
			0	1	2	3	4	5	6	7	8	
4(kHz)	Group	Control	24.5±4.18	24.50±6.12	23.66±6.05	23.66±4.08	24.50±6.12	25.33±6.83	24.50±6.12	26.66±6.37	26.16±4.91	0.94
		Cis7.5	26.16±3.76	28.66±7.52	30.33±8.16	36.16±5.84	37.83±3.76	39.50±5.24	40.33±6.05	40.33±6.05	4.33±6.05	0.00
		Cis10	24.50±2.73	26.16±3.76	28.66±6.05	34.50±6.89	41.16±10.68	44.50±9.35	46.16±7.35	47.00±7.07	47.00±7.07	0.00
		Cis12.5	26.16±2.04	27.83±2.04	29.50±4.18	34.50±5.24	41.16±8.61	62.00±7.74	73.66±6.83	77.00±7.74	79.50±6.89	0.00
8(kHz)	group	control	24.00±3.16	24.83±3.76	24.00±3.16	26.50±6.12	25.66±4.08	26.50±4.18	26.50±4.18	25.66±5.16	26.50±5.24	0.79
		Cis7.5	24.00±4.47	26.50±6.89	28.16±5.84	34.83±3.76	35.66±4.08	37.33±2.58	38.16±3.76	38.16±3.76	38.16±3.76	0.00
		Cis10	23.16±3.76	24.83±3.76	26.50±2.73	32.33±5.16	39.00±4.47	43.16±4.91	45.66±5.16	46.50±5.24	42.83±9.70	0.00
		Cis12.5	24.83±4.91	26.50±5.24	28.16±3.76	34.00±4.47	41.50±2.73	62.33±5.16	73.16±2.04	77.33±5.16	76.33±5.53	0.00
12(kHz)	group	control	17.83±4.91	16.16±5.84	17.00±7.07	16.16±5.84	17.83±3.76	15.33±2.58	16.16±5.84	15.33±6.83	17.00±8.36	0.97
		Cis7.5	17.00±5.47	18.66±4.08	21.16±4.91	27.00±5.47	29.50±4.18	31.16±4.91	32.00±4.47	32.00±4.47	32.00±4.47	0.00
		Cis10	17.83±2.04	19.50±2.73	21.16±3.76	27.00±4.47	35.33±4.08	40.33±5.16	41.16±5.84	42.83±9.70	42.83±9.70	0.00
		Cis12.5	19.50±2.73	21.16±3.76	22.83±3.76	28.66±5.16	37.00±6.32	58.61±7.52	70.33±5.16	75.33±5.16	76.33±5.53	0.00
16(kHz)	group	control	8.83±4.91	9.66±2.58	9.66±4.08	10.50±5.24	9.66±2.58	10.50±4.18	10.50±4.18	10.50±6.89	11.33±4.08	0.97
		Cis7.5	8.83±3.76	10.50±2.73	12.16±3.76	18.83±5.84	21.33±4.08	23.00±4.47	23.83±3.76	23.83±3.76	23.83±3.76	0.00
		Cis10	8.83±4.91	10.50±2.73	12.16±3.76	18.00±6.32	26.33±8.75	32.83±4.49	33.50±5.57	33.83±6.64	33.83±6.64	0.00
		Cis12.5	9.66±4.08	11.33±2.58	13.83±2.04	18.83±7.35	26.33±8.16	48.83±12.41	60.50±7.58	65.50±8.21	67.16±5.84	0.00

## Discussion

A wide spectrum of unpleasant effects on the inner ear, including hearing loss, tinnitus and vertigo have been reported for some drugs, such as cisplatin (26). For many years, researchers all over the world have sought to recognize the basis and pattern of the inner ear impairment in an effort to propose some strategies for the prevention and control of side effects. Cisplatin-induced hearing loss, apart from studies on patients who receive this drug(27), is investigated in numerous experimental animals, such as rats(28,29) and guinea-pigs(17,30,31). According to these studies, cisplatin induces dose-dependent bilateral sensorineural hearing loss(32). In the present study, three different doses of this drug (7.5, 10, and 12.5 mg/kg) were used as daily injections of 2.5 mg/kg in guinea-pigs for 3, 4, and 5 consecutive days to identify the detailed template of the damage. Daily threshold changes of ABR were used as a functional marker for cochlear impairment. As illustrated in Figure1, the total dose of 7.5 mg/kg cisplatin has induced a mean threshold shift of 14.65 dB in four evaluated frequencies (4 kHz, 8 kHz, 12 kHz, and 16 kHz). With increasing the administered dose to 10 and 12.5 mg/kg, the mean threshold shift values reached 24.16 dB and 56.16 dB, respectively. This finding is consistent with the previously published results verifying the cumulative essence of cisplatin-induced hearing loss. Liba et al. (2017) observed median ABR threshold changes of 15 dB by single low-dose cisplatin treatment (8mg/kg). The changes in ABR thresholds are comparable to our observed changes in mean ABR thresholds by using the total dose of 7.5 mg/kg cisplatin (33). Xiong et al. (2011) investigated the role of nitric oxide in cisplatin ototoxicity. In the mentioned study, 72 h after the injection of 10 mg/kg cisplatin in guinea pigs, 22.3 dB and 26.8 dB changes were observed in mean ABR thresholds at 8 kHz and 16 kHz tones, respectively (34). Using the same dose of the drug in guinea pigs, very similar results were obtained, including a 23.33 dB shift in mean ABR thresholds in 8 kHz and 25.83 dB changes in 16 kHz. Murphy et al. (2011) carried out a study to investigate the role of intratympanic dexamethasone in the prevention of cisplatin-induced hearing loss. They indicated mean ABR threshold shift of 23.4 dB by total dose of 10 mg/kg and 57.2 dB shifts by total dose of 12 mg/kg cisplatin in guinea pigs(35). On the other hand, the administration of the single dose of 12mg/kg cisplatin in guinea pigs resulted in 55 dB shifts at 8 kHz tone and 51 dB shift at 16 kHz tone, in the study conducted by Waissbluth et al. (2012)(36). There are a number of articles in this category with nearly the same results confirming the cumulative effects of multiple-low dose IP injection on the hearing system. Nevertheless, it is noteworthy that there is no linear relationship between the total dose of the drug and the resulted hearing loss. In other words, by increasing just one low-dose injection from 10 mg/kg to 12.5 mg/kg, the changes in the hearing threshold remarkably increase. In the present study, we evaluated and recorded the resultant daily increase in mean ABR thresholds in order to exactly analyze the template of these increases and obtain some valuable results. As depicted in Figure 2, the patterning of the threshold change is the same in all groups until the 3^rd^ day of experiment. In group Cis7.5, which received the drug up to the 3^rd^ day, the diagram of threshold elevation grows by a gradual slope in the next days. In group Cis10, there is another rising step almost identical with the recent increases; thereafter, the graph rises almost as slightly as the Cis7.5 group at the 4^th^ day of experiment. In group Cis12.5, following the last injection on the 5^th^ day, there is a sharp surge in the graph demonstrating an upward trend during the next days. This trend leads to a total threshold change of 56.16 dB in mean ABR thresholds in 72 h after the last injection on the 8^th^ day. From a clinical perspective, finding such a momentous dose could be of great help in governing the ototoxicity symptoms. Previous studies have reported that above a certain critical cumulative dose of the drug in the blood, the destruction increases very rapidly. This occurs as the result of permanent renal damage leading to the accumulation of the drug in the blood (37).

The results of the current study highlighted the importance of daily evaluation of the auditory system in patients who receive this drug. The recognition of the template of this degeneration could be of significant importance for the avoidance of undesirable side effects of this drug. On the other hand, daily monitoring of the hearing system is definitely an inexpensive and cost-effective strategy, as compared to the adverse effects of hearing loss on patients who receive the drug, especially children. It is worth mentioning that in humans, the exact pattern of induced hearing loss and the dose of the drug which causes ototoxicity is not as identical as it is in experimental animals(38). 

## Conclusion

On a final note, in the light of the obtained findings of the present study and the results reported in the literature, it can be concluded that the recognition of the template of this degeneration could be of significant importance for the avoidance of undesirable side effects of this drug on hearing system, especially the hearing loss.
